# Supplementary material for: Differential Effects of the Processed and Unprocessed Garlic (Allium sativum L.) Ethanol Extracts on Neuritogenesis and Synaptogenesis in Rat Primary Hippocampal Neurons
Source: Int J Mol Sci. 2023 Aug 29;24(17):13386. doi: 10.3390/ijms241713386 (PMC10487397; doi:10.3390/ijms241713386)
Supplement: Supplementary file 1 [file ijms-24-13386-s001.zip › Supplementary File S1.pdf]

Table S1: Identification of bioactive compounds in 95% ethanol extracts of processed garlic (BGE) with varying levels (%) by GC-MS

RT (Min)	Name of Compound	Peak Area (%)
3.2	1-[(1-OXO-2-PROPENYL)OXY]-2,5-PYRROLIDINEDIONE	7.831667
3.5	SILANE, TRIMETHYLOCTADECYL-	0.411753
4.2	BENZENE, 1,3-DIMETHYL-	8.724239
4.8	BENZENE, 1,3-DIMETHYL-	2.511214
4.025	L-ALANINE, N-METHOXYCARBONYL-, TRIDECYL ESTER	3.213776
6.14	2,6,6-TRIMETHYL-2-CYCLOHEXENE-1,4-DIONE	0.948419
6.74	3-(1'-PYRROLIDINYL)-2-BUTANONE	5.33735
7.27	DODECANE, 1-FLUORO-	0.734376
7.79	2H-PYRAN, 3,4-DIHYDRO-	3.258881
8.85	DODECANE, 2,2,11,11-TETRAMETHYL-	0.138589
9.3	DECANE, 2-CYCLOHEXYL-	0.25123
9.17	LINALOOL	14.59833
9.78	TRIFLUOROMETHYL T-BUTYL SULFIDE	11.61691
11.2	2,3-DIMETHYL-UNDEC-1-EN-3-OL	1.858725
12.7	OCTADECANE, 2,2,4,15,17,17-HEXAMETHYL-7,12-BIS(3,5,5-TRIMETHYLHEXYL)-	2.322186
14.9	DODECANE, 1-FLUORO-	0.492988
14.78	HENTRIACONTANE	1.373999
16.07	1,2,6-HEXANETRIOL	1.262995
19.7	CYCLOPROPANE, 1,1,2-TRIMETHYL-3-(2-METH YLPROPYL)-	0.538974
19.55	UNDECANE, 3,6-DIMETHYL-	0.84415
23.9	SULFUROUS ACID, 2-ETHYLHEXYL HEXYL ESTER	1.496747
28	OCTADECANOIC ACID, 9,10-DICHLORO-, METHYL ESTER	0.892865
38.2	SILANE, DIMETHYL(2,2,2-TRICHLOROETHOXY)TRIDECYLOXY-	0.955453
39.67	CYCLODODECANEMETHANOL, 1-METHOXY-	1.939168

Table S2: Identification of bioactive compounds in 95% ethanol extracts of unprocessed garlic (WGE) with varying levels (%) by GC-MS

RT (Min)	Name of Compound	Peak Area (%)
4.8	BENZENE, 1,3-DIMETHYL-	3.788802
4.29	BENZENE, 1,3-DIMETHYL-	16.82087
6.09	BENZENE, 1-ETHYL-2-METHYL-	0.112775
9.17	LINALOOL	0.715504
9.84	TRIFLUOROMETHYL T-BUTYL SULFIDE 158	0.81643
10.53	2-ETHYLTHIOLANE, S,S-DIOXIDE	0.459682
11.9	2-TRIMETHYLSILYL-1,3-DITHIANE	0.185404
11.38	GLUTARIC ACID, HEX-5-EN-1-YL 8-CHLOROOCTYL ESTER	0.858606
12.72	1,3,4-THIADIAZOL-2-AMINE, 5-METHYL-	0.338646
16.25	METHANE, ISOTHIOCYANATO-	0.417036
22.09	TRIDECANE, 2,2,4,10,12,12-HEXAMETHYL-7-(3,5,5-TRIMETHYLHEXYL)-	8.603642
29.09	DODECANOIC ACID	8.14
30.88	2-ISOTHIOCYANO-2,4,4,6,6-PENTAMETHYLHEPTANE	0.23
31.5	14-METHYLPENTADEC-9-ENOIC ACID METHYL ESTER	0.179037
31.77	12,15-OCTADECADIENOIC ACID, METHYL ESTER	0.237864
32.6	6-OCTADECENOIC ACID	4.812488
32.85	12,15-OCTADECADIENOIC ACID, METHYL ESTER	31.12708
34.17	I-PROPYL 11,12-METHYLENE-OCTADECANOATE	2.245516
34.38	METHYL 5,11,14-EICOSATRIENOATE	8.178718
35.5	GLYCIDYL PALMITATE	6.511326
39.3	ADIPIC ACID, BUTYL CIS-NON-3-ENYL ESTER	0.302635

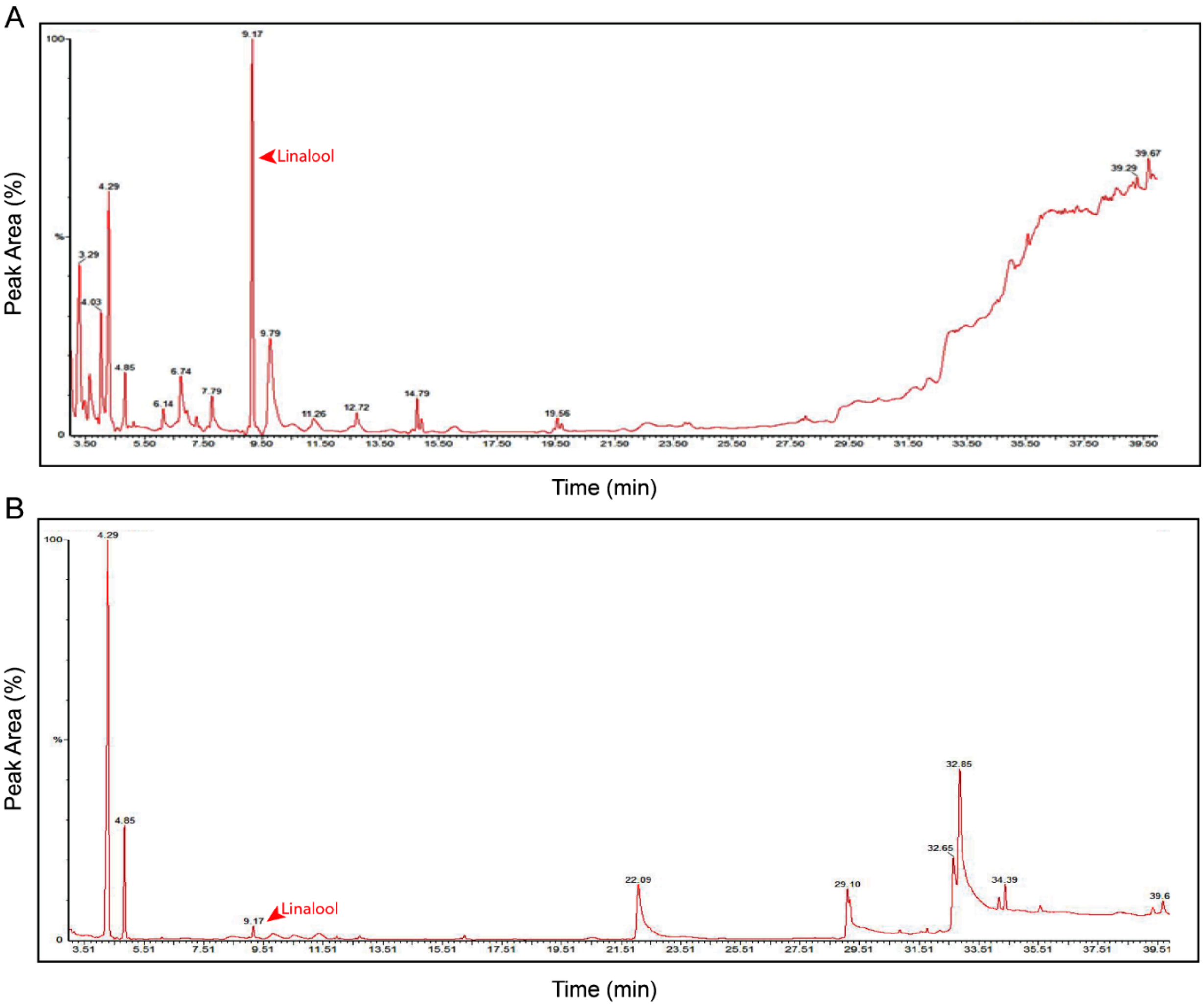

Figure S1: GC-MS chromatogram showing retention time and peak area of different bioactive compounds of BGE (A) and WGE (B).

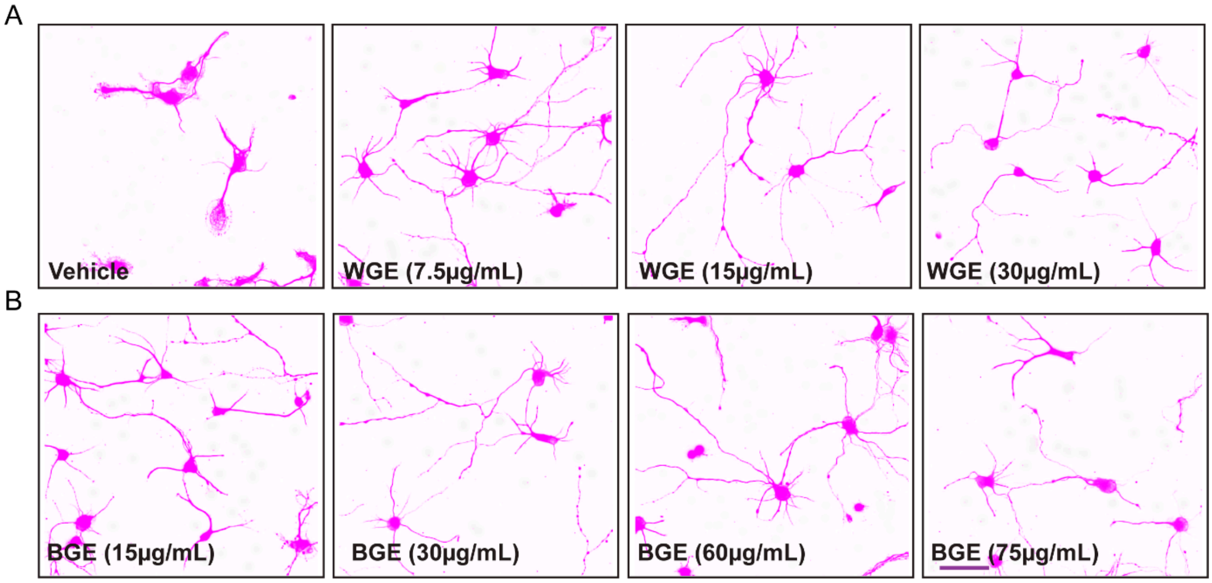

Figure S2: Processed and unprocessed garlic facilitates neuritogenesis in a dose-dependent manner. (A) Representative images showing neurites outgrowth of WGE and (B) BGE-treated neurons for 3 days in a dose-dependent manner compared to the vehicle by α-tubulin staining, Immuno-stained images were inverted and presented as a grayscale mode with pink neurite path and white background; scale bar: 50 µm.

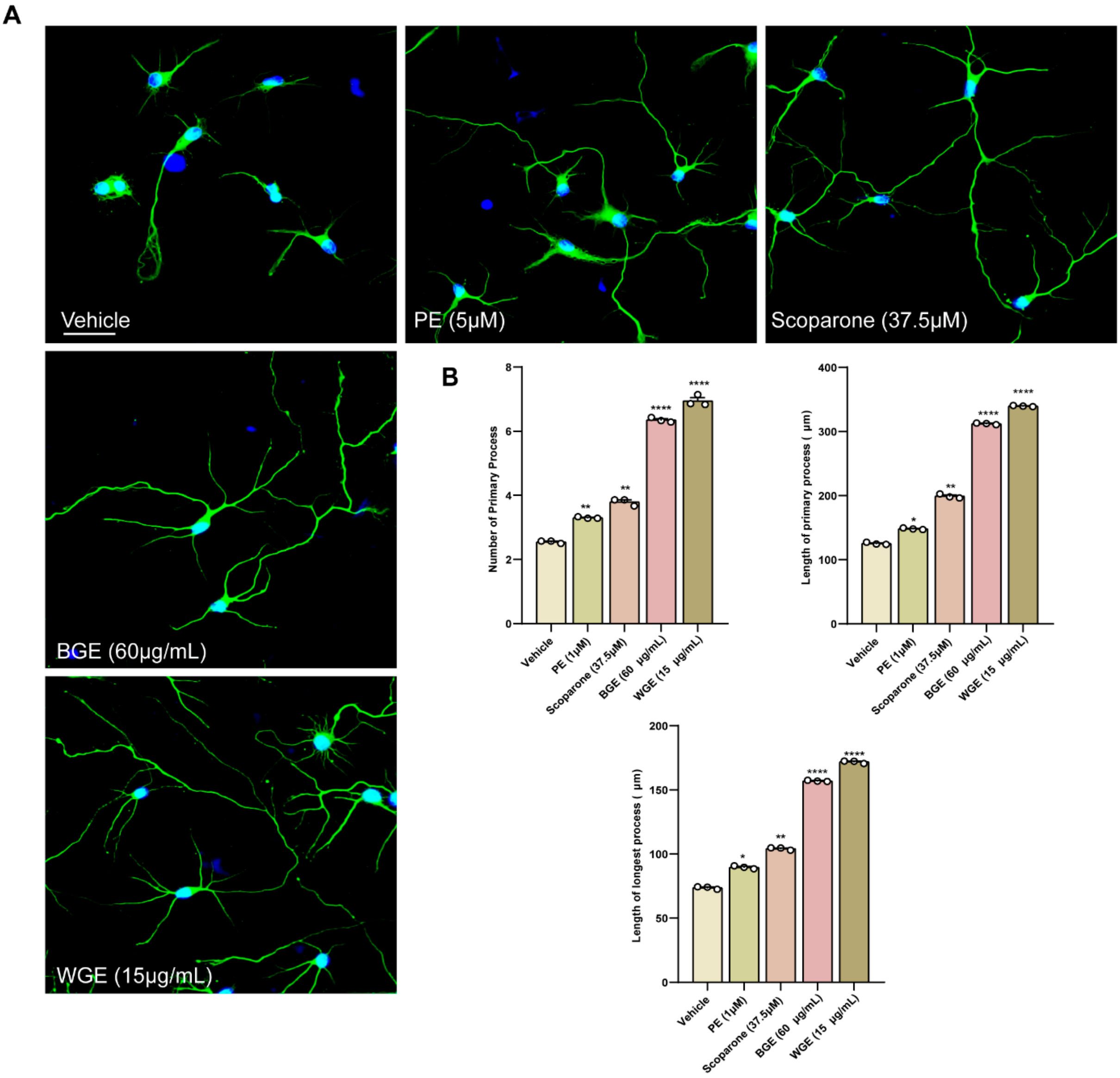

Figure S3: (A) Representative fluorescence images showing the neuritogenic effect of the puerarin (PE) (5 µM), scoparone (37.5 µM), BGE (60 µg/mL), and WGE (15 µg/mL) vs. vehicle immune-stained by α-tubulin (green) with the blue-stained nucleus. scale bar: 50 µm. (B) Calculation of the morphometric study using statistical methods, including the number and length of the total neurites, as well as the length of the longest neurite. Statistics show as mean ± standard error of the mean (S.E.M.) of three separate experiments (n=3, each includes 10 neurons, one-way ANOVA, *$p < 0.05$, **$p < 0.01$, , ****$p < 0.0001$).

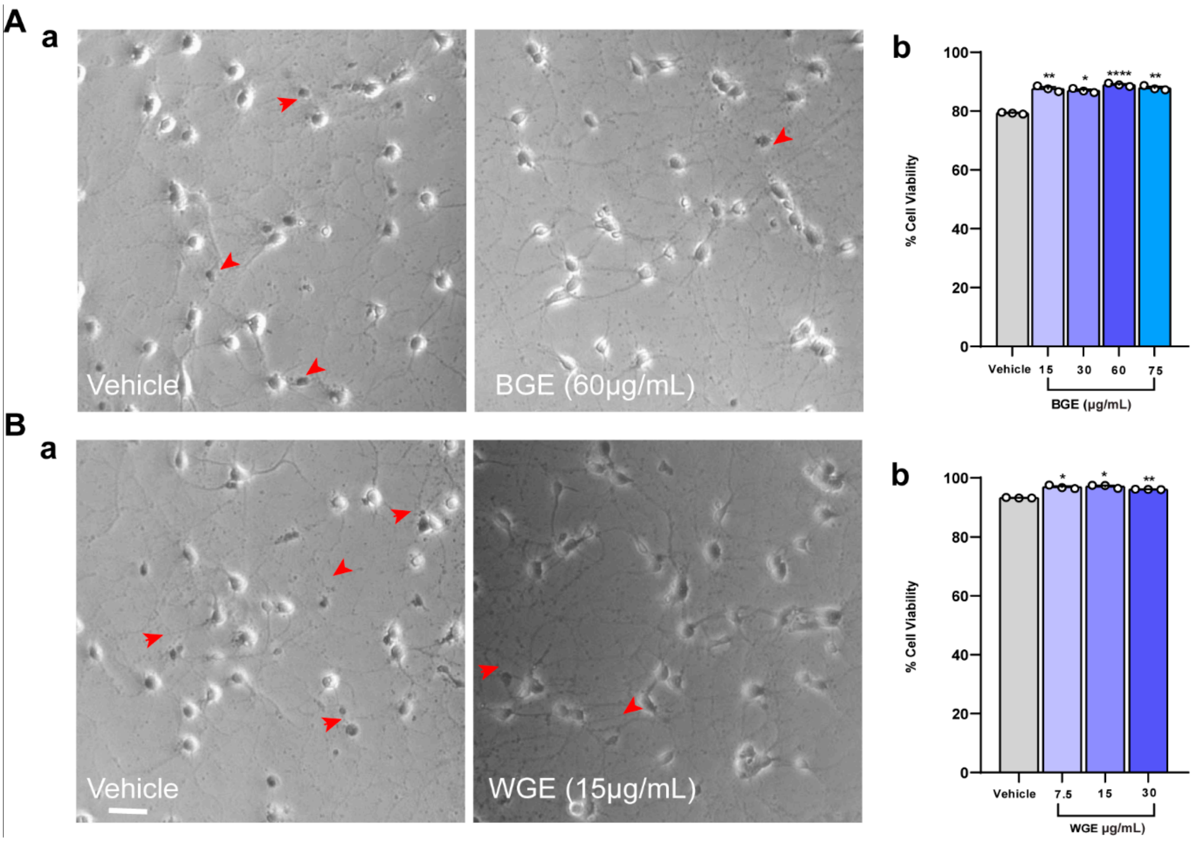

Figure S4: The effects of the BGE and WGE on neuronal cell viability. (A) Trypan blue staining images showing the viable neurons where dead neurons are represented (red arrows) (a), bar graph showing the percentage of survival cells treated with BGE (60 µg/mL) (b). (B) Representative phase-contrast images showing the viable neurons (a), bar graph showing the percentage of survival cells treated with WGE (60 µg/mL) (b); scale bar, 50 µm. Here, the percentage of alive neurons was calculated by counting the number of unstained cells relative to the sum of cells examined (living plus dead). Statistics show as mean ± standard error of the mean (S.E.M.) of three separate experiments (n=4, each includes 500 neurons, one-way ANOVA, *$p < 0.05$, **$p < 0.01$, ****$p < 0.0001$).

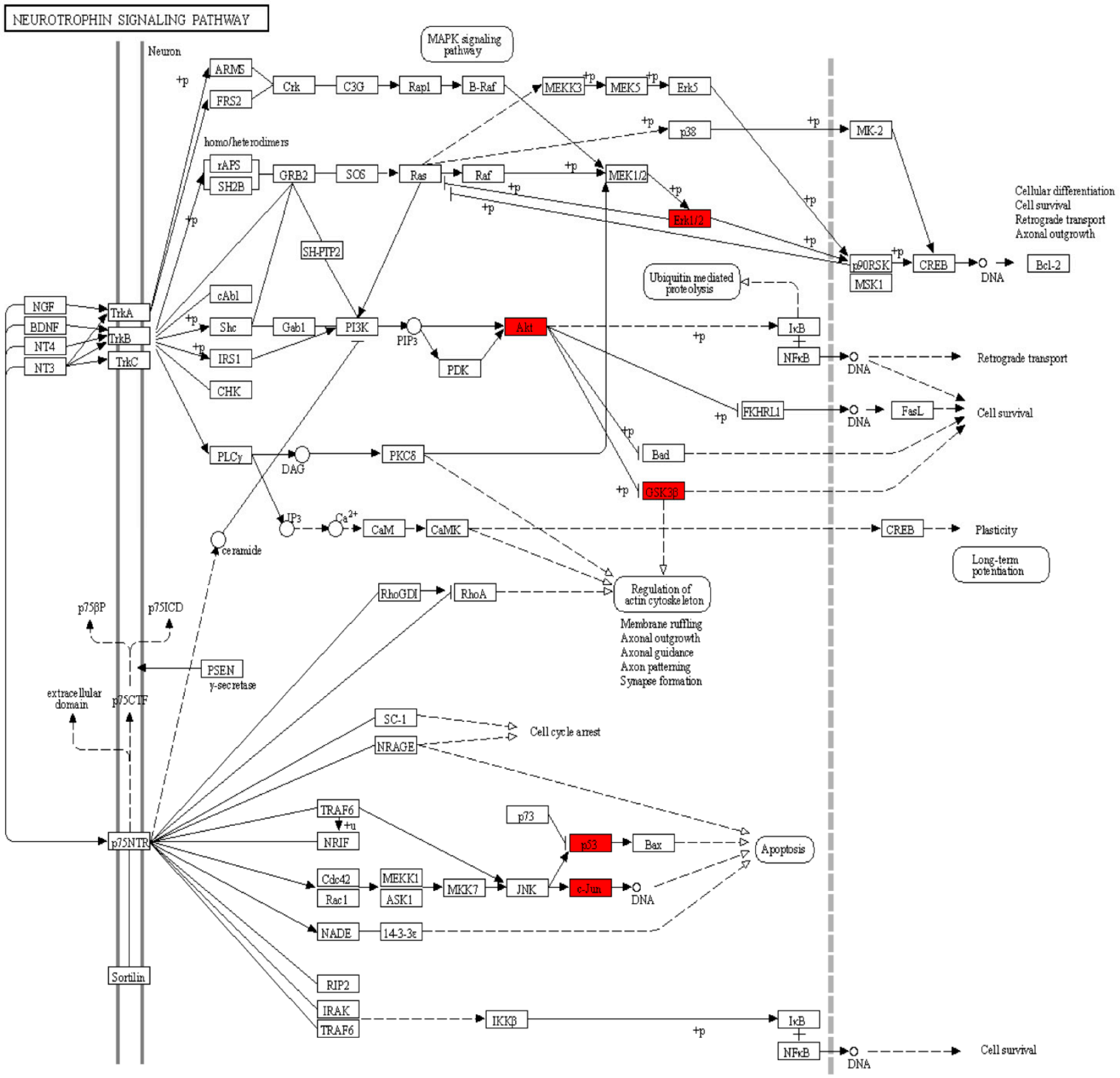

Figure S5: The KEGG pathway analysis uncovered the target's involvement in the neurotrophin signaling pathway of the bioactive component of BGE and WGE. Red boxes represent the identified genes that were modulated by linalool.

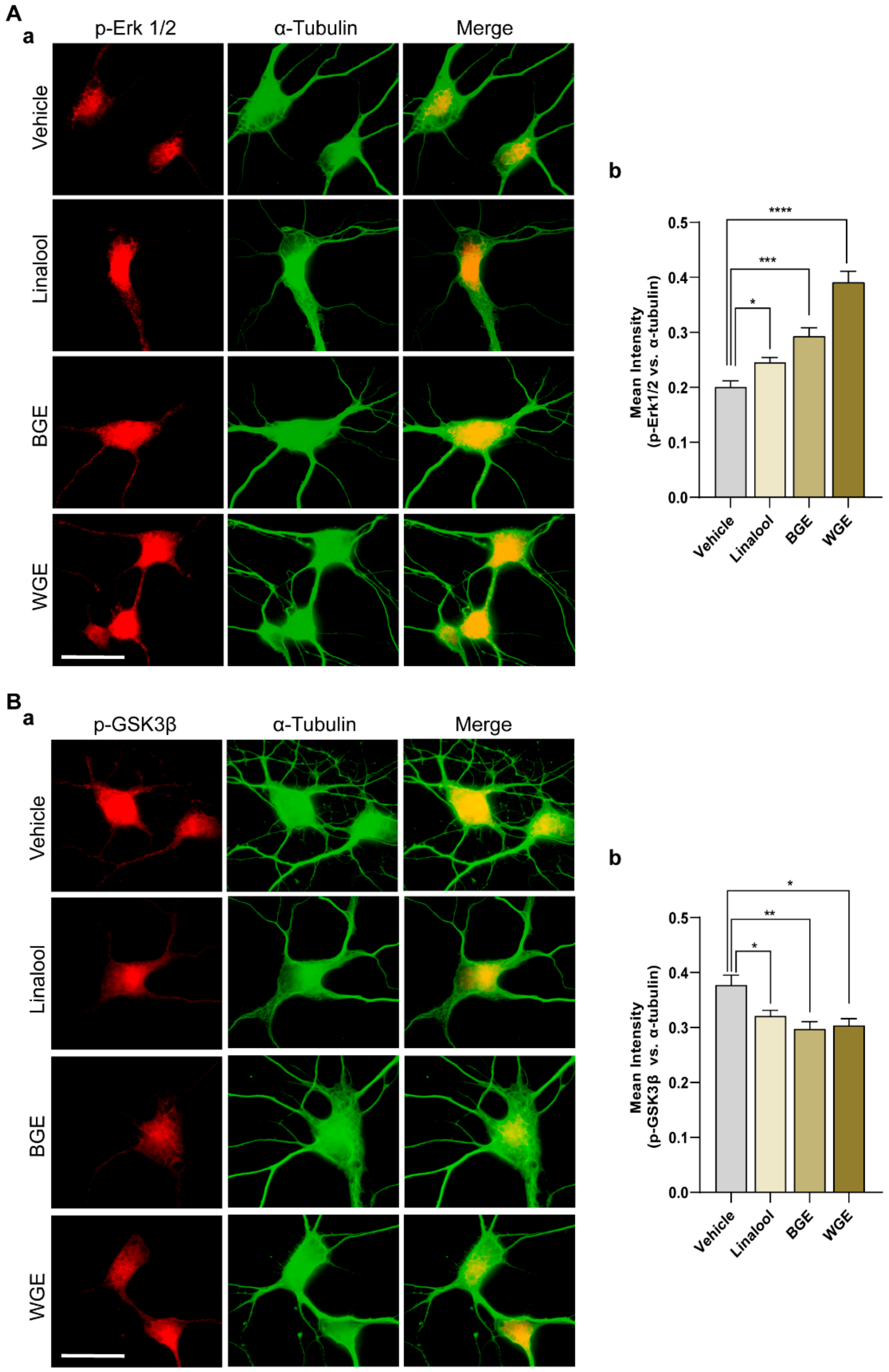

Figure S6: Validation of target genes expression of the neurotrophin signaling system by Immunofluorescence analysis. (A) Representative fluorescent images showing p-ERK1/2 expressions in red and double immuno-stained with α-tubulin in green (a), scale bar: 20μm. Fluorescence intensity ratios of P-Erk1/2 vs. α-tubulin (b). (B) Representative fluorescent images showing p-GSK3β expressions in red and double immuno-stained with α-tubulin in green (a), scale bar: 20μm. Fluorescence intensity ratios of p-GSK3β vs. α-tubulin (b). Statistics show as mean ± standard error of the mean (S.E.M.), one-way ANOVA, *p < 0.05, **p < 0.01, ***p < 0.001, ****p < 0.0001).
